# Using verbal and social autopsy approaches to understand why neonates die in rural settings: a case study of a remote rural district in Uganda

**DOI:** 10.1136/bmjph-2023-000682

**Published:** 2024-04-17

**Authors:** Felix Kizito, Rornald Muhumuza Kananura, Jacquellyn Nambi Ssanyu, Joseph Akuze, Dinah Amongin, Peter Waiswa

**Affiliations:** 1 Department of Health Policy Planning and Management, School of Public Health, Makerere University College of Health Sciences, Kampala, Uganda; 2 Busoga Health Forum, Jinja, Uganda; 3 Newlife Adolescent and Youth Organization, Wakiso, Uganda; 4 School of Public Health, Centre of Excellence for Maternal & Newborn Health, Makerere University, Kampala, Uganda; 5 Maternal & Reproductive Health Unit, Institute of Tropical Medicine, Antwerpen, Belgium; 6 Advance Innovations for Transforming Health, Kampala, Uganda; 7 African Population and Health Research Center, Nairobi, Kenya; 8 Department of Women's and Children's Health, Uppsala University, Uppsala, Sweden; 9 Karolinska Institute, Stockholm, Sweden

**Keywords:** public health, cross-sectional studies, public health practice

## Abstract

**Introduction:**

Neonatal mortality remains a formidable challenge in low-resource settings, such as Uganda, despite global health initiatives. This study employs a social and verbal autopsy approach to identify the causes, settings and health accessibility challenges surrounding neonatal deaths in the Luuka district from 1 January 2017 to 31 December 2019.

**Methods:**

We analysed data from 172 neonatal verbal and social autopsies (VASA) conducted over 3 years, as part of a maternal and neonatal demand and supply health system strengthening intervention. Cause-of-death coding was done by two independent medical officers using WHO-ICD-10 guidelines to ascertain the causes of death. VASA-coded data analysis of the causes of death was done in STATA V.14.0. In addition, 16 key informant interviews were conducted, including 2 community health workers,6 household members and 8 health workers, with qualitative data analysed through thematic content analysis.

**Results:**

Among the 172 neonate deaths, 95.9% occurred in the early neonatal period (0–6 days) and 4.1% in the late neonatal period (7–27 days). The primary causes of death were birth asphyxia (42.4%), low birth weight/prematurity (18.6%), other perinatal causes (12.8%) and neonatal sepsis (9.3%). Delays in getting appropriate care at the facility (delay 3) and delays in seeking care (delay 1) (51.2% and 44.2%, respectively) were linked to newborn mortality. Qualitative insights underscored inadequate awareness of neonatal danger signs, deficient referral systems, drug shortages, reliance on unskilled traditional birth attendants and insufficient neonatal care facilities as significant contributors.

**Conclusion:**

Addressing delays in both home-based care (delay 1) and timely access to appropriate care in healthcare facilities (delays 2 and 3) is pivotal in mitigating neonatal mortality. Comprehensive interventions targeting improved access to maternal services and enhanced quality of care in health facilities are imperative for advancing newborn survival in rural settings.

**WHAT IS ALREADY KNOWN ON THIS TOPIC**

**WHAT THIS STUDY ADDS**

- Multiple factors concurrently affect access to appropriate obstetric and newborn healthcare services.
- Deliveries in community clinics and homes contribute the largest share of neonatal mortality rate.

**HOW THIS STUDY MIGHT AFFECT RESEARCH, PRACTICE OR POLICY**

- Targeted community-based and facility-based quality-of-care interventions are required.

## Introduction

 Globally, nearly 2.3 million neonates succumbed to mortality every year, translating to an alarming approximately 6400 neonatal deaths each day.^1^ Sub-Saharan Africa (SSA) emerges as a focal point in this disconcerting scenario, consistently contributing the largest proportion to the overall global neonatal mortality burden.^1 2^ Particularly noteworthy is the stagnation observed in neonatal mortality rates (NMRs) within the SSA region, persisting at an approximate rate of 27 deaths per 1000 live births for nearly 2 decades.^1^ Such stagnation raises insightful concerns about the adequacy of current health systems in effectively delivering requisite interventions to those in need.

Despite the well-established efficacy of cost-effective interventions in preventing neonatal mortality, the persistent stagnation in SSA over the past two decades suggests a potential failure in the health systems to adequately reach and provide appropriate interventions to the target population. Addressing this critical issue is imperative for meeting the ambitious Sustainable Development Goal and the commitment of countries to reduce the NMR to no more than 12 per 1000 live births.^3^

Addressing these supply-side deficiencies and fortifying the healthcare workforce are imperative steps towards enhancing the overall efficacy of health systems in Uganda and, by extension, accelerating progress in reducing NMR.^3^

In nations characterised by weak civil and vital registration systems, exemplified by Uganda, the utilisation of verbal autopsy (VA) and social autopsy (SA) (VASA) has emerged as an essential method for exploring the intricate web of social, cultural and health system factors contributing to NMR.^4 5^ The dual objectives of SA underscore its significance: first, furnishing healthcare programmes and policy-makers with population-level data to inform the development of more efficacious strategies, newborn care programmes and technologies; second, fostering awareness of maternal and child deaths as preventable issues, thereby empowering communities to actively engage with health programmes, enhancing responsiveness and fostering accountability.^6 7^

In situations where a medically certified cause of death is unavailable, VA serves as an invaluable indirect method for ascertaining the biomedical cause. This involves extracting information on symptoms, signs and circumstances preceding death from deceased caretakers.^5 8^ However, to derive programmatically relevant insights, it is imperative not only to ascertain how a newborn perished but also to understand why SA, as a method, assumes a pivotal role in unravelling the complexities of the ‘why’.^1 9^

Regrettably, the implementation of VASAs at the population level during programme implementation remains infrequent, despite their potential to inform health systems strengthening endeavours. Notably, no district-wide VASAs have been conducted in Uganda during newborn programming in any district, highlighting a notable gap in leveraging these methodologies to enhance neonatal care. Moreover, the scarcity of data poses a considerable challenge in comprehending the reasons behind neonatal deaths, even within the context of health system strengthening initiatives.^10^ This gap is particularly pronounced in SSA, where a significant proportion of deaths occur at home without a documented cause.^2^ In such circumstances, the judicious application of VA and SA methodologies becomes imperative to expose the reasons behind persistent high NMR and contribute meaningfully to informed health interventions and policy formulations.

we employed a comprehensive approach, integrating both VA and SA approaches to examine the causes of neonatal deaths in connection with the delays in accessing newborn health services, where the newborn deaths take place, and delays in accessing appropriate newborn healthcare services. The study was conducted between 1 January 2017 and 31 December 2019, within the geographical areas of Luuka district in eastern Uganda.

**Figure 1:**
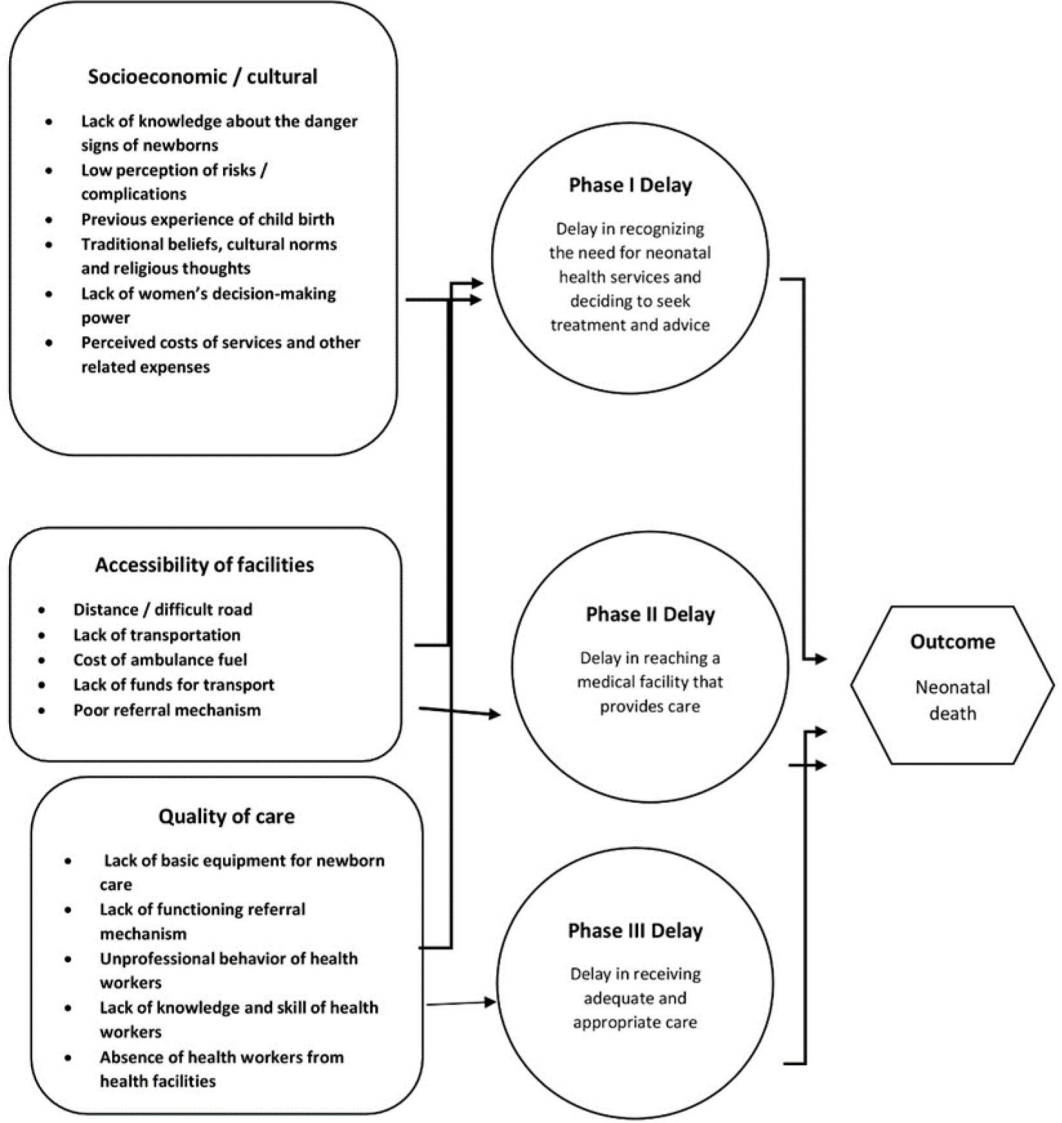
Theoretical framework.

Our analysis was guided by a three-delay model^11^ and we modified it to understand how a range of social and health facility factors affect access to appropriate healthcare services(figure 1). The three-delay model, which consists of delay 1 (delay in deciding to seek care), delay 2 (delay in reaching the facility) and delay 3 (delay in receiving care), has been overwhelmingly used to evaluate the circumstances surrounding access to adequate emergency obstetric care.^12 13^ Even though to assess neonatal deaths, the most preferred method is the pathway to survival framework, we preferred to use the three-delay model to understand the different delays and how they can also contribute to neonatal deaths.^14^

## Methods

### Study design and area

The study was a cross-sectional study using both VA and SA approaches. This study was conducted in the Luuka district. Luuka is a ‘new’ district that was carved out of the Iganga district. It is bordered by the districts of Kaliro in the North East, Iganga to the South East, Mayuge to the South, Jinja to the South West and Kamuli in the North West. The district has one county (Health sub-district) with seven rural subcounties and one town council. It contains 43 parishes and 281 approved village councils with a population of 238 020 people of which 124 454 (52.3%) are females and 113 566 are males. At a growth rate of 2.1%, a fertility rate of 7.3% and an average household size of 9.3, the district population grew from 185 526 in 2002 to 2 38 020 in 2014. Of this population, 61.1% is below 18 years.^15^

The provision of basic health and social services is majorly a responsibility of the government and its development partners through the district health and social service delivery mechanism. The district has no hospital; one health centre (HC) IV, 8 HC IIIs and 20 HC IIs (which provide outpatient care). The HC III facilities provide antenatal, delivery and postnatal services. HC IV facility levels provide emergency obstetric care and newborn services; it has a neonatal room to offer treatment for newborns with health emergencies and an operational theatre.

In Uganda, the health system is decentralised into the district health team and the central ministry of health. The district should have a general hospital, HC IV at each health subdistrict which supervises HC IIIs which often give maternal services in addition to ambulatory care below HC IIIs are dispensaries labelled HC IIs. At the community level, there are community health worker (CHWs) (level 1) who provide day-to-day referrals to patients from the community.^16^

### Study population and sampling

The target populations consisted of neonates who died within 28 days after delivery between the times of 1 January 2017 to 31 December 2019 and were born within the Luuka district, CHWs, facility health workers and caretakers in homesteads. The neonatal deaths must have been reported by the CHWs in the communities of the Luuka district and assessed using the VASA tool.

### Quantitative methods

#### Data collection procedures

The study used VASA data collected by Community in which Mothers and New born Thrive project-trained research assistants. Training of five research assistants was done by the community field coordinator and medical officer in English. These research assistants were at the graduate level of education. The trained research assistants visited homes where deaths occurred as reported by the CHWs in their death notification monthly reports. During VASA data collection, interviews were done with mothers of deceased neonates or the immediate care in cases where both mother and newborn passed on. A recall period of 1–6 weeks was applied. The interviewer took the interviewee through a series of questions in quest to generate adequate information surrounding the events of the death of the neonate, and the underlying circumstances, occurring during the care-seeking process. This data included the deaths of neonates and mothers which happened at the facility and community levels. The VASA tool used was adopted from the World Health Organisation (WHO) VASA collection tool. There was a translation of the questions into the local language of the research area for easy interpretation of the questions by the interviewer. A bar of soap was given to the homestead visited by research assistants as a token of appreciation for their time. After VASA data collection by the project, community and district feedback was given, respectively

#### Data management and analysis

Two research assistants at a graduate level were trained in the entry of coded data in Excel and also in collecting and summarising discussion and interview information in Microsoft Word. VASA data was entered into Microsoft Excel and exported into STATA V.14.0 for analysis.

Data that were reviewed by two independently trained medical doctors (FK and WJ) were analysed for each death to ascertain the cause of death using WHO-International Classification of Diseases (ICD)-10 guidelines.^4 17^ During the review, any disagreement between the two reviewers, there was a discussion and if an agreement was reached on the diagnosis, it was accepted as the definite cause of death. In cases where an agreement was not reached the cause of death was coded as undetermined. The coded data were entered into Excel, cleaned and exported into STATA V.14.0 for descriptive analysis of quantitative data concerning causes and contributing factors.

### Qualitative methods

For qualitative data, purposive sampling was used for the CHWs, health workers and caretakers. Two CHWs, eight health workers and six mothers or immediate caretakers (in cases where both mother and neonate passed on) were held to get more understanding of the contributing factors to neonate deaths within the Luuka district. A representative sample using a rule of saturation was used. Stillbirths were not considered for this study. This was a population-based survey that looked at all neonatal deaths with VASA collected in the period of 1 January 2017 to 31 December 2019.

#### Data collection procedures

An interview guide was designed with questions based on the objectives of the study to collect qualitative data. All audio interviews were audio recorded. The interview guide was pretested to ensure that it captured the required information. Results from this process were not included in the final results. The pretest was done to adjust and inform the final tool for the qualitative interviews. The interviews were conducted in English for health workers. Translation into local languages was done by CHWs. This was transcribed back into English by the facilitator and note-taker. All the audio recordings were transcribed by an experienced research assistant. Mothers who had lost their neonates were counselled and empathy was shown throughout the interview process. Furthermore, they were referred to see a professional counsellor for free when the need arose. All efforts were made to ensure data completeness. A revisit of the family was made in cases where some information was missed on the form.

#### Data management analysis

The interviews were recorded, and at the same time, they were translated and transcribed by using Microsoft Word. A comparison of notes with the audio was made to ensure consistency of information. Each recording was individually listened to and transcription was done in Microsoft Word verbatim. We manually analysed the data thematically using a deductive approach with a semantic-level focus in Microsoft Excel. During the deductive thematic analysis, some of the codes developed were care, transport, facility, home, traditional birth attendant (TBA) and equipment to the transcribed data (codes were brief descriptions of what was being said in the interview). Then codes were sorted into subthemes and then themes. Direct quotations were used in the presentation of the study findings. There was masking of the study participants’ identity.

Direct quotations were used in the presentation of the study findings. There was masking of the study participants’ identity. Information given in table 5 is some of the comments made by mothers whose homesteads were visited regarding neonatal deaths, health workers at the facility and CHWs attached to particular facilities during key informant interviews. We identified subthemes that were knowledge gaps of mothers and health workers, beliefs and practices, decision-making power of women, cost of services and transportation gaps. We reviewed the themes and refining was done to ensure no contradictions and overlaps occurred within the themes. In cases of contradictions within the theme, splitting was done into separate themes. A description of each theme was done in Microsoft Word. The themes were social economic/cultural determinants, accessibility of facilities and quality of care.

### Reflexivity

During data collection, we were aware of how the professional backgrounds of the two reviewers, both medical doctors, might shape the research process. While their medical expertise could enhance understanding of terminology and biomedical intricacies in VA data, contributing to analytical rigour, there was a potential for this perspective to overshadow the broader sociocultural factors. Recognising this, we were sensitive to the social setting, conducting interviews in participants’ workplaces or homes, allowing them control over the pace, and deliberately adopting a ‘back seat’ approach to empower participants and maintain a sense of control over the interview process. The team was also committed to constant comparison during data analysis for a comprehensive exploration of participants’ accounts.

### Patient and public involvement

There was no involvement of the patient and the public in this research.

## Results

### Social demographics

Table 1 shows the social demographic of health workers, CHWs and mothers interviewed during KI interviews. Five of the eight midwives interviewed were enrolled midwives while three were registered midwives. These midwives had a history of working in maternity wards, especially in HC III and IV. The average years of experience of working in the district were 9.5 years with the highest years of experience being 29 and lowest 2 years. Six of these midwives were married while two were single. The mothers’ average age was 26 years with the oldest at 32 and youngest at 20 years of age. The majorities were staying in semipermanent homes and did peasant farming as the main economic activity. The average age of their neonates who passed on was a day old. Most of these mothers were at the primary level of education and were married.

**Table 1:** Demographics of health workers (HWs), community health workers (CHWs) and mothers/caretakers interviewed

Participant no.	Position	Age	Department	Marital status	Level of education	Facility level	Years of service
HW1	Registered midwife	51	Maternity	Married	Diploma	H/C IV	29
HW2	Enrolled midwife	28	Maternity	Married	Certificate	H/C III	5
HW3	Enrolled midwife	28	Maternity	Married	Certificate	H/C IV	8
HW4	Enrolled wife	25	Maternity	Single	Certificate	H/C IV	2
HW 5	Enrolled midwife	30	Maternity	Married	Certificate	H/C III	4
HW 6	Registered midwife	27	Maternity	Single	Diploma	H/C III	7
HW7	Enrolled midwife	30	Maternity	Married	Certificate	H/C III	8
HW 8	Registered midwife	35	Maternity	Married	Diploma	H/C III	13

Participant no	Position	Age	Attached department at the facility	Marital status	Level of education	Level of health centre	Years in service
CHW1	CHW	50	Maternity	Married	Secondary	HC IV	20
CHW 2	CHW	43	Maternity	Married	Secondary	HC III	10

		Caretakers/mothers’ demographics					
Subcounty	Age	Gravidity	Age of baby	Level of education	Marital status	Occupation	Condition of home
Bukanga	29	04	00	Married	Secondary	Business	Permanent
Waibuga	20	02	01	Married	Primary	None	Semipermanent
Bukanga	29	05	00	Married	Primary	Peasant	Mud and wattle
Bukanga	21	01	01	Married	Primary	Peasant	Semipermanent
Bukanga	32	05	01	Married	Primary	Peasant	Semipermanent
Waibuga	25	06	02	Married	Primary	Peasant	Semipermanent

### Descriptive characteristics of the neonates

Among the 172 neonatal deaths reviewed, 54.1% were male and 45.9% were female. In table 2, most of the deliveries of the neonates occurred in government HCs at 44.8%, 36% at private clinics, 13.4% of these neonates were delivered at home, 4.1% of the deliveries occurred at TBA homes, whereas 1.7% of the births occurred on the way to the health facility. However, most neonate deaths occurred in private health facilities (32%) and public facilities (31%), 25% of the neonate deaths occurred at home, 9.3% of these deaths occurred before reaching the health facility (on route to facility) and 2.3% occurred at the TBA’s home.

**Table 2:** Showing place of birth and death of neonates

Place of birth/death	n/N (%) births	n/N (%) deaths
Private facility	62/172 (36)	55/172 (32)
Government centre	77/172 (44.8)	54/172 (31.4)
TBA	7/172 (4.1)	4/172 (2.3)
Home	23/172 (13.4)	43/172 (25)
On route to facility	3/172 (1.7)	16/172 (9.3)

TBAtraditional birth attendant

**Figure 2:**
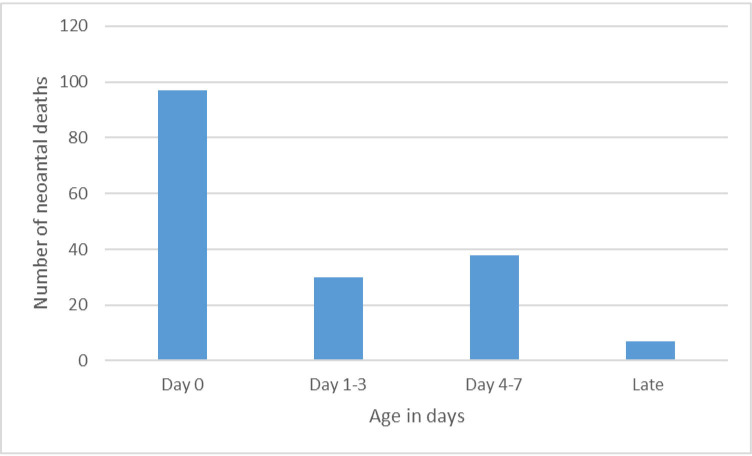
The distribution of deaths by age.

The number of babies who died in the early neonatal period was 95.93% (n=165) and the late neonatal period was 4.07% (n=7). Most neonates died in the early neonatal period as depicted by the figure below. The majority of these deaths occurred at day 0 and day 4 after birth. In the late neonate period, deaths occurred more at day 14 (figure 2).

### Causes of neonatal death

Birth asphyxia was the top three causes of death accounting for 42.4% of deaths, low birth weight/prematurity at 18.6% and neonatal sepsis (9.3%) (table 3).

**Table 3:** Summary of the immediate causes of neonatal deaths (n=172)

Cause	n (%)
Birth asphyxia	73 (42.4)
Low birth weight and/or prematurity	32 (18.6)
All perinatal causes	22 (12.8)
Neonatal sepsis	16 (9.3)
Anaemia	9 (5.2)
Pneumonia	9 (5.2)
Undetermined	3 (1.7)
Congenital abnormalities	2 (1.2)
Haemorrhagic shock	2 (1.2)
Syphilis infections	2 (1.2)
Sudden baby death syndrome	1 (0.6)
Diarrhoeal diseases	1 (0.6)

### Delays contributing to neonatal deaths

The three-delay model was used in the analysis of delays associated with neonatal deaths. Delay to getting appropriate care at health facility (delay 3) contributed to 51.2% (n=88) of neonatal deaths (table 4). Closely followed, 44.2% (n=76) of deaths were contributed by delay in making decision to seeking care (delay 1). 4.7% (n=8) were due to delays in reaching the health facility (delay 2).

**Table 4:** Showing the delays and associated barriers of neonatal mortality (n=172)

Delayn (%)	Associated factors
Delay 1(delay in recognising the need for neonatal health services and deciding to seek treatment and advice)76 (44.2)	Traditional beliefs, culture norms and religious thoughts^15^Poor perceptions of costs of services and other related expenses.^10^Low perception of risks/complications.^18^Delayed to make decision to come to facility^22^Failure to recognise danger signs^7^Poor coordination and communication among the different providers^17^
Delay 2 (delay in reaching a medical facility that provides care)8 (4.7)	Lack of transport to referral point^11^Long distance to the hospital^2^Poor referral mechanism.^3^
Delay 3 (delay in receiving adequate and appropriate care)88 (51.2)	Lack of basic equipment for newborn care^40^Unprofessional behaviours of health workers/negligence^17^Lack of knowledge and delivery skills by the health worker^31^Lack of functional referral mechanism^12^

The information given in table 5 is some of the comments made by mothers whose homesteads were visited in regard to neonatal deaths, health workers at the facility and CHWs attached to particular facilities during key informant interviews.

**Table 5:** Theme and subtheme of the key informant interview (KII) on the causes and delays of neonatal deaths in the Luuka District, Eastern Uganda

Theme	Subtheme	Details
Social economic/cultural determinants (delay 1)	Home deliveries	‘I ended up delivering at home’—M1-KII
	Lack of knowledge about the benefits of maternal health and danger signs.	‘I just heard that bleeding during pregnancy is bad’—M1-KII
	Low perception of the risk/complication	‘I didn’t know that the situation would worsen’—M2-KII
	Traditional beliefs, cultural norms and religious thoughts (concerning pregnancy, burial of placenta, trust in TBA.	‘I was told that I will be able to deliver well at that place because she has been delivering other women well’—M2-KII
	Lack of decision-making power of women.	‘My husband decided that we go to that place in Busalaama’—M2-KII
	Perceived poor attitude of the health workers.	
	Perceived cost of healthcare services	‘It’s not possible to go to the health facility without money because there is nothing for free’—M2-KII
	Cultural beliefs and practices	‘We used some local medicine when the labor started’—M2-KII
Accessibility of facilities	Lack of transport.	‘We find it hard get a motorcycle that would take us at night to the health facility’—M3-KII
	Long distances to health facilities	‘The health facilities where the deliveries occur are very far’—M3-KII
	Poor roads	
Quality of care	Inadequate knowledge of the health workers.	‘Cannot do Nozzle gastric tube feeding for babies’—(HW-KII Bukanga)
	Lack of essential drugs and supplies at the health facility	‘No protocols and guidelines in the management of sick newborns, Power blackout for Oxygen supply, No cylinder heads, Stock outs of drugs’—HW-KII Bukanga
	Poor referral systems	‘Its VHTs that refer mothers with complications’—HW-KII BukangaFor most newborn complications we just refer
	Unprofessional behaviour of the health workers	‘Doctor not available to handle emergencies’’—HW-KII-Kiyunga‘Unsolicited referrals to higher levels’—HW-KII Kiyunga
	Lack of knowledge and skills of health workers	‘No knowledge on the MPDSR, No committees in place’—HW-KII-Bukoova
	Absence of skilled health workers at the health facility	‘Health workers not skilled in the management of sick newborns’—CHW-KII-Ikonia‘Long waiting time at the health facilities’—CHW-KII Irongo

TBAtraditional birth attendant

### Delay 1 causes

Some of the major factors contributing to this delay were (1) delay in decision-making to come to facility and (2) low perception of the risks.

#### Delay in decision-making to come to facility

Mothers experienced delays in making the decision to seek medical assistance, often due to the need for spousal consent. Many indicated that they would consult with their husbands before deciding to go to a healthcare facility. If the husband perceived no urgent need for medical attention, the mother would choose to stay home. Additionally, they mentioned a tendency to visit traditional healers prior to considering a visit to the healthcare facility.

For instance, one participant shared her experience, stating, ‘*My husband was the one who suggested that we visit the place in Busalaama [the traditional healer]’* (Participant M2-KII).

#### Inadequate knowledge/low perception of newborn danger signs

A notable concern identified was the insufficient knowledge among mothers regarding the potential danger signs in newborns. From the qualitative interviews, mothers lacked awareness of critical indicators of newborn health. Tragically, there was a reported incident where a newborn lost its life due to bleeding through the umbilical cord.

One healthcare worker shared an illustrative example, stating*, ‘In some cases, a baby might experience bleeding from the cord, and the mother might not recognize the severity of the situation. This oversight can result in the baby developing anaemia, ultimately leading to a tragic outcome’* (Key Informant Interview - Healthcare Worker).

A healthcare worker shared their insights, remarking, ‘*Despite the availability of government healthcare facilities, some individuals continue to resort to local herbal remedies and unskilled TBA deliveries. This choice often exposes mothers and newborns to unsanitary conditions and limited resources’* (Key Informant Interview—Healthcare Worker).

### Delay 2 causes and associated factors

Although this delay played a comparatively minor role in neonate deaths, it remained a concerning issue. The primary contributors to this delay included:

#### Lack of transport means

Despite the presence of motorcycles, a common mode of transportation for many mothers, financial constraints often prevented them from accessing these services. This financial burden hindered movement and subsequently led to delays in seeking necessary care, particularly exacerbated during nighttime due to the remote setting. Notably, the district was served by only one ambulance at the time of the study. One mother stated that ‘*We find it hard get motorcycle that would take us at night to the health facility’—M3-KII*

#### Long distance to the hospital

It was noted that low level facilities are situated at a considerable distance from higher level facilities, creating a notable challenge for transportation. For example, Iganga Hospital, which is the nearest higher-level facility, is located 30 km away. This distance presents a significant obstacle for individuals trying to access medical care. Coupled with the poor condition of roads, this distance contributed to delays in reaching the health facility promptly. One mother shared her concerns stating*, ‘The health facilities where the deliveries occur are very far’—M3-KII’’*

#### Poor referral systems

Instances were reported where mothers with complications were referred to higher-level facilities for advanced care. Unfortunately, these referrals often lacked clear explanations regarding the necessity and urgency. Delays were common between the referral initiation and arrival at the designated referral site. A healthcare worker acknowledged the complexity, stating, ‘*There are times when health workers contribute to delays, especially when we're hesitant to refer a mother who is not progressing, leading to foetal distress’* (Key Informant Interview—Healthcare Worker).

### Delay 3 causes

There were two main factors associated with delay in receiving quality and appropriate care (1) lack of basic equipment for newborn care and (2) lack of knowledge and delivery skills by health workers.

#### Lack of basic equipment for newborn care

##### Out of stock of drugs and other supplies for sick newborns

A significant concern highlighted is the alarming shortage of essential drugs and supplies required for the effective management of newborn complications. Surprisingly, interviewed health workers from healthcare facilities within the district reported a lack of access to vital supplies and medications essential for addressing newborn care complications. Notably, this shortage was more pronounced in lower-level health facilities, which were often inadequately constructed and lacking the necessary infrastructure for managing newborn complications.

A healthcare worker shed light on the situation, stating, ‘*We are facing critical shortages of essential equipment. Currently, we lack a resuscitation table and essential protocols for managing newborn complications, including essential newborn care, Helping Babies Breathe, addressing jaundice, providing appropriate feeding for small infants, and promoting kangaroo care’* (Key Informant Interview—Healthcare Worker 1).

##### Lack of space for care for sick newborns

An overarching challenge pervading the health facilities within the district is the severe constraint on available space, especially given that most of these facilities operate at lower levels. This shortage of adequate space is further compounded by an insufficient number of human resources for health. This scarcity has implications for the comprehensive care of newborns and their mothers. Healthcare workers who participated in the study highlighted that the lack of adequate space and resources often leads to the referral of cases to higher-level facilities, primarily due to the absence of sophisticated medical equipment that requires more room.

A healthcare worker provided insight into this dilemma, explaining, ‘*We grapple with a space shortage, particularly concerning practices like Kangaroo care. Ideally, a separate room or space is needed, but the current situation sees postnatal mothers, malaria cases, and abortion cases all crammed into the same room due to space constraints’* (Key Informant Interview—Healthcare Worker 1).

##### Lack of knowledge and skills by the health workers to manage sick newborns

Front-line healthcare providers, notably midwives and nurses, have revealed a concerning deficiency in their technical competence for managing newborn complications. This inadequacy is attributed to a dearth of proper training and skills development. A notable portion of midwives disclosed that they lacked orientation and training in effectively managing newborn complications.

One healthcare provider candidly shared their experience, stating, ‘*There are instances when we encounter newborn complications that we feel ill-equipped to handle. In such situations, we often rely on peer-to-peer support for guidance on treating these complications. If a colleague or friend is unavailable, I've even reached out to midwives in other areas like Kiyunga for advice’* (Key Informant Interview—Healthcare Worker 1).

## Discussion

The study identified critical challenges within the healthcare system leading to delays in seeking and providing appropriate care for newborns. Key themes included delays in decision-making to seek care, scarcity of essential drugs and supplies, limitations in healthcare facility infrastructure and inadequate technical competence among front-line healthcare providers.

Delays in accessing appropriate newborn healthcare services were linked to low perception of risks, traditional beliefs and financial constraints. Transportation-related factors, particularly the lack of quality transportation options, emerged as a major contributor to delays in reaching healthcare facilities.

Even on reaching healthcare facilities, delay 3 (delay in getting appropriate care) was observed due to limited equipment, the absence of essential medicines and incompetence among health workers, especially in private and lower-level facilities.

The primary causes of neonatal deaths in the rural community were birth asphyxia, low birth weight/prematurity and neonatal sepsis. Delays in care-seeking, inadequate transportation and deficiencies in healthcare facility quality were identified as major contributing factors.

The majority of neonatal deaths occurred in private facilities, emphasising the need for improved quality control, licensing and inspection in the private healthcare sector.

The first aim of our study was to examine the delays in accessing appropriate newborn healthcare services. Delay in deciding to seeking care and reaching the healthcare point (delay 1 and delay 2) as a result of low perception of the risks plus traditional beliefs affected health seeking behaviours of the mothers and caretakers of the neonates. Similar findings were reported in studies conducted in Uganda, Rwanda and Ethiopia, which revealed that delay one was still among the major contributing factor of neonatal death.^2 5 12^ Studies done in Ghana and Nigeria show that there is limited knowledge and decision-making autonomy of mothers in rural settings concerning pregnancy and neonatal health emergencies.^6 7^ It is indicative of a critical gap in the understanding of danger signs, exacerbating the challenges faced by mothers in seeking timely and appropriate care.^7^

Significantly, delay in reaching the healthcare point (delay 2), which is rooted in transportation-related factors, emerged as a contributor to the aforementioned delays. Studies done in India, Ethiopia, Nigeria and Uganda’s show that transport related delays are a significant contributor to neonatal deaths.^27^,^9 12^ Instances where mothers or caretakers had to contend with waiting for transportation or faced unavailability of means at the time of need further compounded the challenges in accessing healthcare facilities or referral points. These findings are comparable to those of a system review study done in Africa and other similar research.^2 10^

Furthermore, our investigation revealed that even on reaching healthcare facilities, the situation was compounded by delay 3 (delay in getting appropriate care while at facility), primarily attributable to limited equipment, the absence of essential medicines crucial for neonatal care and incompetence among health workers notably in private and lower-level facilities. These findings are in line with studies done in Rwanda, Uganda where the shortage of essential drugs, supplies and equipment emerged as a significant barrier to effective newborn care.^2 5^

Our findings revealed a concerning gap in the technical competence of front-line healthcare providers in managing newborn complications. These findings are consistent with research conducted in Uganda, Ethiopia, Rwanda and India.^2 5 9 12^ This inadequacy often led to seeking peer support or even consulting colleagues from other facilities for guidance. Insufficient training and skills development left midwives and nurses ill prepared to handle critical situation. High-quality antenatal care, skilled care at birth, postnatal care for mothers and baby care of small and sick newborns are all recommended by international policies.^1^

Our study also highlighted the suboptimal state of healthcare facility infrastructure, lack adequate space which hinders the provision of specialised care such as Kangaroo care. These findings are similar to studies done in eastern Uganda and Kenya.^2 18^ The inadequate physical environment, coupled with the shortage of skilled healthcare professionals, further compounded delays in providing appropriate care.^19^ These findings highlight systemic deficiencies within the healthcare infrastructure that hinder effective intervention and exacerbate neonatal health outcomes.^20 21^

The secondary objective of our study was to scrutinise the causes of neonatal deaths. In this study, birth asphyxia was a predominant cause of neonatal mortality within this rural community. These findings are not different from studies done in similar settings where birth asphyxia is still among the top causes of neonatal deaths.^2 22^ Neonates who survive asphyxia at birth have high chances of developing neurological complications including epilepsy, cerebral palsy and developmental delays.^23^,^26^

Low birth weight/prematurity came in second as a cause of death in these data, which is unsurprising given the vulnerability of such babies, as well as what is known in literature.^27 28^ In this scenario, factors such as a shortage of room in facilities to handle preterm newborn and a lack of knowledge and skills among health staff to handle preterm babies posed a challenge for improved management of such babies. These are similar challenges in a study done in Ethiopia.^19^

Additionally, we identified neonate sepsis common cause of neonate deaths. To our understanding, this could be attributed to practices like applying of non-recommended substances like local herbs on the babies’ cords and poor hygiene of the cord. The attribute of sepsis to neonatal death was lower compared with other studies done in Eastern Uganda.^2^ Neonatal sepsis if poorly managed may result into long-term complications such as neurodevelopment impairment.^29^ ^30^ In addition to hygiene during the delivery process and in the facility, environment is critical since illness can transit from facility I to the newborn.^31^,^33^

Lastly, the third objective of our study was to examine where the newborn deaths took place. It was noted that the majority of the deaths occurred in private facilities. Some mothers would rather go to private facilities than government facilities for healthcare. This is due to closeness to the facilities, improved health workers attitude and guaranteed health workers presence. Even though mothers seek support from the private facilities, there is no guarantee of the quality of care provided. ^34^ A study done in India comparing neonatal death between private and government facilities showed that the risk dying in early neonatal period were even higher for babies delivered in private clinic than government facilities.^35^

TBAs are still conducting deliveries within this rural district despite various efforts to curb the practice. The distances to the facility, low education level and harsh/poor attitude from the health workers all contributed to this. This is in line with findings from previous research conducted in Uganda and Kenya.^36^,^38^ Such deliveries put the life of the mother and newborn at greater risk of death however if they are trained and supported by authorities, studies have shown that they can play a crucial role in reduction of maternal and newborn deaths.^39^,^41^

### Strengths and limitations of the study

The biggest strength of this study lies in the use of VASA, an approach that allows a comprehensive understanding of the multifaceted challenges in accessing appropriate newborn healthcare services. Through VASA, the study explored diverse aspects, considering social, cultural and healthcare system factors, providing nuanced insights into the complexities faced by mothers, health workers and the community. Thematic analysis of the qualitative data also enriched the study by revealing distinct perspectives on barriers to newborn healthcare, contributing to a profound understanding of the challenges in care-seeking

Nevertheless, we acknowledge the limitations within the study. Care-seeking information and illness history were based on interviews with non-medical persons, which introduces a potential source of bias in the information gathered. Additionally, the sampling frame for VASA data was limited to only 172 deaths which occurred within 3 years, which possibly limits the generalisability of the findings beyond this period. Interviews depending on recall also pose reliability and validity problems. However, it is noteworthy that severe symptoms tend to be remembered more accurately than mild ones. Moreover, the effort to conduct most interviews within 4–6 weeks of death minimised the recall bias.

### Implications for policy and practice

The study underscores the interconnected challenges in newborn care, emphasising the need for a holistic and collaborative approach from healthcare authorities, policy-makers, communities and healthcare professionals.

Comprehensive maternal and community education, strengthening healthcare facility infrastructure, ensuring drug and equipment availability, and specialised training for healthcare providers are crucial policy and practice implications.

Improving transportation options, particularly in rural contexts, and enforcing strict laws on TBAs are essential steps to address delays and reduce neonatal mortality.

### Priorities for future research

Future research should focus on further analysing neonatal deaths in private facilities, conducting comparative investigations in Luuka district and exploring the effectiveness of interventions aimed at reducing delays and improving neonatal care.

Research on the impact of cultural and social dynamics on care-seeking behaviours, as well as the effectiveness of community engagement through CHWs, could provide valuable insights.

Continued examination of healthcare infrastructure, quality control in private facilities, and the impact of maternal and community education programmes on care-seeking behaviours should be prioritised for a comprehensive understanding and sustainable interventions.

### Conclusion

This study underscores the multifaceted nature of delays in newborn care within the district. The identified challenges are interconnected and addressing them requires a holistic and collaborative effort from healthcare authorities, policy-makers, communities and healthcare professionals. To address these challenges and mitigate delays in newborn care, a multipronged approach is warranted. First and foremost, there is an urgent need for comprehensive maternal and community education to raise awareness about the importance of timely care-seeking and the dangers of relying solely on traditional remedies. Strengthening healthcare facility infrastructure, ensuring the availability of essential drugs and supplies, and investing in specialised training for healthcare providers are crucial steps towards improving the quality of newborn care. By addressing delays at both the community and facility levels, comprehensive interventions can significantly contribute to reducing neonatal mortality in these settings.

## Data Availability

All data relevant to the study are included in the article or uploaded as online supplemental information.
